# Clarification on the implementation of the Nagoya Protocol in France for the access and sharing of benefits arising from the utilization of microbial genetic resources

**DOI:** 10.1099/ijsem.0.006262

**Published:** 2024-03-06

**Authors:** Mariana L. Ferrari, Olivier Chesneau, Dominique Clermont, Praveen Rahi, Michel-Yves Mistou, Perrine Portier, Fay Betsou

**Affiliations:** 1 Institut Pasteur, Université Paris Cité, Biological Resource Center of Institut Pasteur – Project Management Office, F-75015, Paris, France; 2 Institut Pasteur, Université Paris Cité, Biological Resource Center of Institut Pasteur – Collection de l’Institut Pasteur, F-75015, Paris, France; 3 Université Paris-Saclay, INRAE, MaIAGE, 78350 Jouy-en-Josas, France; 4 Univ Angers, Institut Agro, INRAE, IRHS, SFR QUASAV, CIRM-CFBP, Angers, F-49000 Angers, France

**Keywords:** ABS, access and benefit sharing, France, legislation, microbial resources, Nagoya Protocol

## To the editor

Researchers working with genetic resources (GRs) from France and wishing to validly publish new prokaryotic species names have been facing challenges similar to those encountered by colleagues working with GRs from other countries that are Parties of the Nagoya Protocol on Access and Benefit Sharing (ABS) and that have also decided to regulate the access to their GRs. Such regulations may include different levels of restrictions to the access to GRs. With this letter we aim to clarify some points of the French ABS system that may have created misunderstandings relative to what could be perceived as a restriction, in particular regarding the non-commercial utilization of GRs and the access to French prokaryotic type strains. Moreover, we aim to support the International Committee on the Systematics of Prokaryotes and the *International Journal of Systematic and Evolutionary Microbiology*, and the scientific community at large, in their work with French GR.

Since the adoption of the Nagoya Protocol in October 2010, and its entry into force on 12 October 2014, 140 countries have become Party. The Protocol establishes an international framework for the access to GRs and their associated traditional knowledge in view of their utilization in research and development, the sharing of benefits arising from the utilization of those GRs and associated traditional knowledge, and the compliance of users of those genetic resources and associated traditional knowledge. France signed the Nagoya Protocol as early as 2011 and ratified it with the French law of 8 August 2016 on reclaiming biodiversity, nature, and landscapes (Loi no. 2016–1087 pour la reconquête de la biodiversité, de la nature et des paysages, www.legifrance.gouv.fr/jorf/id/JORFTEXT000033016237). With this law, a national access and sharing system for the benefits arising from the utilization of French GRs and associated traditional knowledge (ABS system) was introduced, both for in-country and foreign-hosted French GRs. The French ABS system is applied to GRs from mainland France and its overseas territories, and does not cover French Polynesia and New Caledonia, which apply their own ABS regulations on the GR under their sovereignty.

In the case of microorganisms, the French ABS system had implemented a 3-year exemption pilot programme, during which all types of utilizations of microorganisms from mainland France were exempt from ABS procedures (Art. 129 of Law No. 2019–486, https://www.legifrance.gouv.fr/loda/article_lc/LEGIARTI000038497920). This exemption ended on 30 August 2022. Nevertheless, as we explain below, the French ABS regulation should not be considered as a restriction to the access to microbial GRs, in the sense of the Rule 30 of the International Code of Nomenclature of Prokaryotes.

For the sake of clarity, we would like to, first, outline here the procedures to be followed to comply with the general French ABS system. These are detailed at the ABS Clearing-House (https://absch.cbd.int) and vary according to the type of utilization of French GRs:

- For *non-commercial utilization*, an ABS declaration (https://absch.cbd.int/en/database/ABSCH-PRO-FR-249352-6) by the user is required (via form CERFA 15786 www.formulaires.service-public.fr/gf/getAnnexe.do?cerfaAnnexe=1&cerfaFormulaire=15786*02) for all new access after 8 August 2016. The declaration process is quite straightforward and once the declaration is submitted, a receipt corresponding to an IRCC (Internationally Recognized Certificate of Compliance) is provided. Importantly, this “receipt” corresponds to an acknowledgement of reception by the French national competent authority, and not to a “permit”, which could be denied to the declarant. Moreover, the declaration can be done retrospectively if a utilization of a GR has taken place by a user, unaware of this procedure.
- For *commercial utilization* (https://absch.cbd.int/en/database/ABSCH-PRO-FR-249353-6) and *utilization of traditional knowledge associated with GR* (https://absch.cbd.int/en/database/ABSCH-PRO-FR-249354-4), however, ABS authorizations are required (via forms CERFA 15785 https://www.formulaires.service-public.fr/gf/cerfa_15785.do and CERFA 15784 https://www.formulaires.service-public.fr/gf/cerfa_15784.do, respectively). The *new utilization of a GR***,** defined as “any research and development activity with a direct commercial development objective and for which the activity area stands out from the initial one of the same user with the same genetic resource or associated traditional knowledge” (Art. L. 412–6), also requires an ABS authorization.

While the perpetuation of the exemption for the utilization of microbial GRs is under debate, the access and utilization (either commercial or non-commercial) of indigenous microorganisms is now regulated by the French ABS system as follows, according to the French law and to the French National Focal Point response:

1. The utilization of certain GR does not require to follow any ABS procedure (Paragraph I Art. L. 412-5) and no “receipt” from the national focal point is to be delivered. This is particularly the case for domesticated or cultivated species (https://agriculture.gouv.fr/telecharger/136842); model species (https://www.legifrance.gouv.fr/jorf/id/JORFTEXT000039180351/); GR collected by laboratories for the prevention and surveillance of diseases or food risks; GR collected by reference laboratories (i.e. the French National Reference Centres); human genetic resources; human microbiota.
2. The following activities involving microorganisms do not require any ABS action at all and no “receipt” from the national focal point is needed, since they are not considered as “utilization”:
  - Acquisition and maintenance of GR in collections.
  - Transfer or sale.
  - Deposit in collections.
  - Access for identification/taxonomy purposes.
3. For non-commercial utilization, an ABS declaration is needed for all new access after the 8 August 2016 only when genetic and/or biochemical analyses are performed on microbial GRs with the aim of increasing the amount of knowledge about the characteristics of a strain/species or the properties of its genes, going beyond the mere identification of a new microbial species (as defined in the guidance document on the scope of application and core obligations of European Regulation (EU) No 511/2014 on the compliance measures for users from the Nagoya Protocol on Access to Genetic Resources and the Fair and Equitable Sharing of Benefits Arising, https://eur-lex.europa.eu/legal-content/EN/TXT/?uri=celex%3A52016XC0827%2801%29). *This is a simple declaration made by the user and not a request for authorization*. No declaration is required for utilisation of GRs that had been accessed before the cited date.

In summary, and as explained above, the currently applicable procedure implemented by the French government for the non-commercial use of microorganisms involves a simple declaration and delivery of an IRCC “receipt”, that does not hinder in any way the access to GRs, including of type strains, while valuing traceability and transparency on utilization of GRs and biodiversity.

*Reviewed and approved by the French ABS National Focal Point, Ministère de la Transition écologique et de la Cohésion des territoires*

